# Family expansion and gene rearrangements contributed to the functional specialization of PRDM genes in vertebrates

**DOI:** 10.1186/1471-2148-7-187

**Published:** 2007-10-04

**Authors:** Irene Fumasoni, Natalia Meani, Davide Rambaldi, Gaia Scafetta, Myriam Alcalay, Francesca D Ciccarelli

**Affiliations:** 1 Department of Experimental Oncology, European Institute of Oncology, IFOM-IEO Campus, Via Adamello 16, 20139 Milan, Italy

## Abstract

**Background:**

Progressive diversification of paralogs after gene expansion is essential to increase their functional specialization. However, mode and tempo of this divergence remain mostly unclear. Here we report the comparative analysis of PRDM genes, a family of putative transcriptional regulators involved in human tumorigenesis.

**Results:**

Our analysis assessed that the PRDM genes originated in metazoans, expanded in vertebrates and further duplicated in primates. We experimentally showed that fast-evolving paralogs are poorly expressed, and that the most recent duplicates, such as primate-specific *PRDM7*, acquire tissue-specificity. *PRDM7 *underwent major structural rearrangements that decreased the number of encoded Zn-Fingers and modified gene splicing. Through internal duplication and activation of a non-canonical splice site (GC-AG), *PRDM7 *can acquire a novel intron. We also detected an alternative isoform that can retain the intron in the mature transcript and that is predominantly expressed in human melanocytes.

**Conclusion:**

Our findings show that (a) molecular evolution of paralogs correlates with their expression pattern; (b) gene diversification is obtained through massive genomic rearrangements; and (c) splicing modification contributes to the functional specialization of novel genes.

## Background

The expansion of gene families in particular evolutionary groups as well as in single lineages significantly contributes to different aspects of genome innovation. However, not all classes of genes undergo equal rate of expansion. Genes controlling basic cellular functions, such as DNA modification, cytoskeletal organization, protein and RNA metabolism are remarkably conserved both in sequence and copy number across large evolutionary distances [1,2]. Other functional categories progressively expand during evolution, with mode and tempo of this expansion largely depending on the effects on organism fitness. Genes coding for structural proteins and those controlling pathogen and stress response usually undergo lineage or organism specific expansions [3,4]. In these cases environment-dependent regulation of gene dosage and expression is required. Genes coding for adhesion molecules, extracellular signalling or those involved in gene regulation expand preferentially during transitions towards higher complexity, defined as increase in cell types [4]. The accretion of Zn-Fingers in vertebrates is among the most positively correlated with increase in complexity [5]. The reason for the massive expansion of Zn-Fingers resides in the ability to bind DNA and regulate gene transcription in a tissue or cell-specific fashion. How this specificity is reached after gene expansion remains still an open question. According to the widely accepted model of evolution by gene duplication [6], in the first phases of their life novel genes are particularly free to change due to relaxed pressure acting on them. The most likely outcome is the acquisition of harmful modifications leading to loss of function (*nonfunctionalization*). However, in very rare cases, novel genes can acquire advantageous mutations differentiating their function from that of the ancestor (*neofunctionalization*). In a third scenario, needed to explain the abundance of gene duplications and lineage-specific expansions, mutations may occur in both genes, ancestor and duplicate, that specialize in complementary functions (*subfunctionalization*) [7-9].

**Figure 1:**
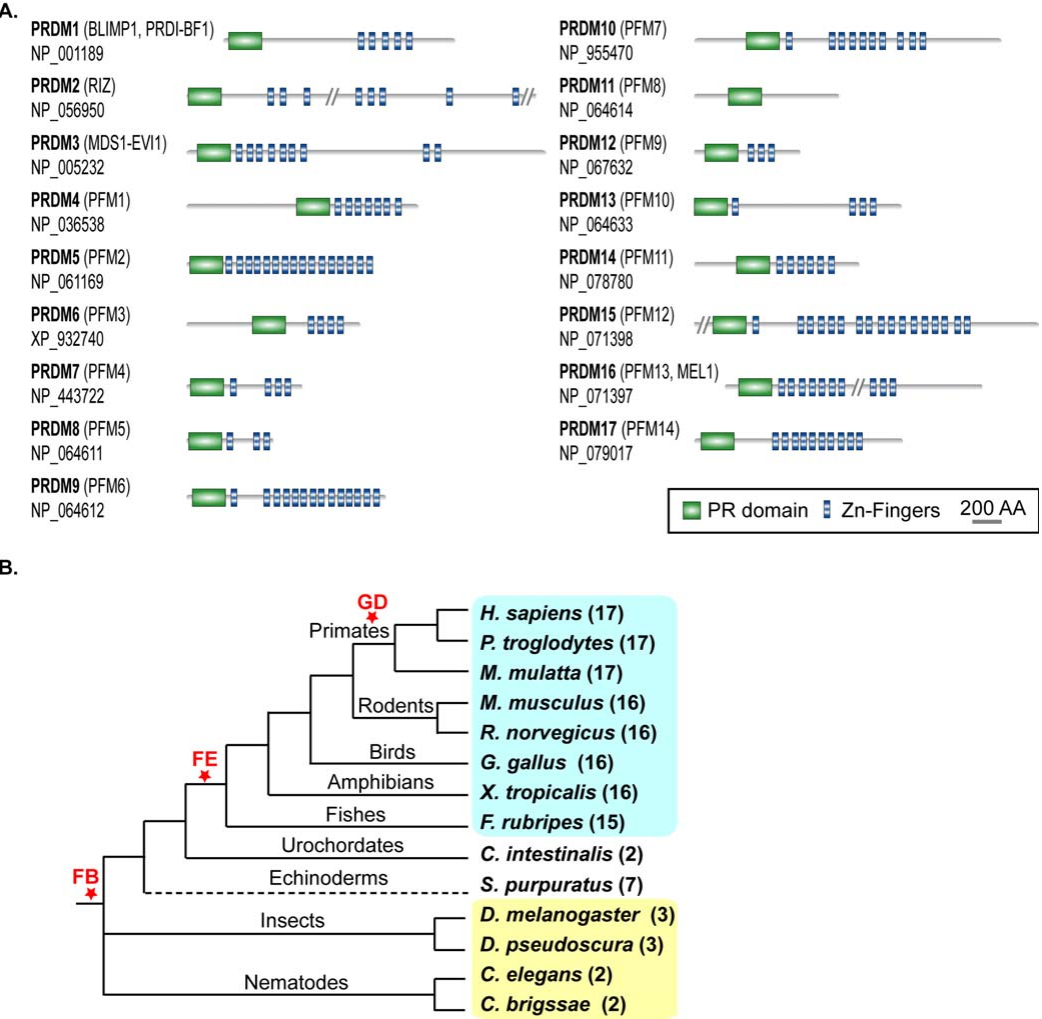
**Domain architecture and phylogenetic distribution of the PRDM proteins**. **(A) **Domain architecture of the human PRDM paralogs. For each PRDM protein, the corresponding RefSeq Accession Number and additional names are provided. Protein regions longer than 1000 amino acids and with no domains are shown as vertical bars (PRDM2, 1239 amino acids; PRDM15, 1507 amino acids; PRDM16, 12769 amino acids). **(B) **Distribution of PRDM genes in representatives of metazoans. For each species, the number of PRDM paralogs is reported in brackets. The species highlighted in cyan are vertebrates, those in yellow are invertebrates. FB, family birth; FE, family expansion; GD, gene duplication.

Here we report a comparative analysis of the PRDM genes in vertebrates with the aim of connecting the expansion of this family of transcriptional regulators to their progressive functional specialization. The results of this study also contribute to unravel the general principles that drive gene family evolution. PRDM genes code for proteins controlling critical aspects of cell integrity, spanning from cell commitment and differentiation [10] to cell growth and apoptosis [11-13]. These genes also play a role in human cancer, where they mainly act as tumor suppressors [14]. PRDM proteins share a characteristic domain organization with an N-terminal PR domain followed by a variable number of Zn-Finger repeats. The only exception is PRDM11, for which no Zn-Fingers are detectable (Figure 1A). The PR (PRDI-BF1 and RIZ) domain is 20–30% identical to the SET module, which is directly responsible for the catalytic activity of several histone lysine-methyltransferases [15]. Some members of the PRDM family show intrinsic methyltransferase activity [16,17], while others play an indirect role by recruiting chromatin remodelling enzymes [18,19]. The number and features of PRDM genes in other species besides human are poorly known. Except for mouse, most PRDM orthologs in other organisms remain uncharacterized. Such lack of information has so far prevented from clarifying the reciprocal evolutionary relationships between different members of the PRDM family.

We started our analysis by searching for orthologs of the 17 human PRDM genes in representatives of fully sequenced eukaryotic genomes. We assessed that, after their appearance in metazoans, PRDM genes expanded in vertebrates and further duplicated in primates. By comparing the evolutionary features of PRDM genes with their expression in human tissues, we detected a tendency of newer genes to acquire tissue specificity. Slowly evolving paralogs also produce more abundant transcripts. We found that the massive structural rearrangements of primate-specific *PRDM7 *gene greatly contributed to modify gene function. In particular, the duplication of an internal segment conferred peculiar splicing pattern to the new gene, with the gain of a novel intron or, alternatively, its retention in the mature RNA.

## Results and discussion

### PRDM genes expanded in vertebrates and further duplicated in primates

By searching for PRDM orthologs in a variety of eukaryotic genomes, we did not identify any PRDM gene either in fungi or in plants, confirming that they first appeared in metazoans. In invertebrates, the orthology assignment yielded 2 genes for nematodes and 3 genes for arthropods (Figure 1B and Additional file 1). The additional sequence found in insects corresponds to *Hamlet*, a transcription factor involved in dendrite morphogenesis [20], and acting as negative regulator of S-phase entry in drosophila [21]. Interestingly, also vertebrates PRDM2 [13], PRDM5 [12], and PRDM9 [16] are involved in cell cycle regulation. Among vertebrates, we identified 17 putative PRDM orthologs in primates and 16 putative orthologs in rodents, birds and amphibians (Figure 1B). In fugu we only detected 15 PRDM genes, as we could not identify the *PRDM17 *ortholog possibly due to the presence of unresolved regions in the genome assembly.

Our results highlighted the expansion of the PRDM family in vertebrates. We tried to assess a possible timing for this expansion by checking for putative PRDM orthologs in protochordates and other invertebrates. We were able to identify only 2 PRDM genes in *C. intestinalis *(Figure 1B), likely because of the poor quality of the ciona genome assembly. The retrieval of 7 putative PRDM orthologs in the echinoderm *S. purpuratus *indicated that at least a partial expansion of the family preceded the last common ancestor of vertebrates (Figure 1B). Our data also showed that PRDM gene expansion continued in primates, as one single rodent co-ortholog corresponds to the primate in-paralog pair formed by *PRDM7 *and *PRDM9 *(see Additional file 1).

### Expression pattern of PRDM genes reflects their evolutionary relationships

**Figure 2:**
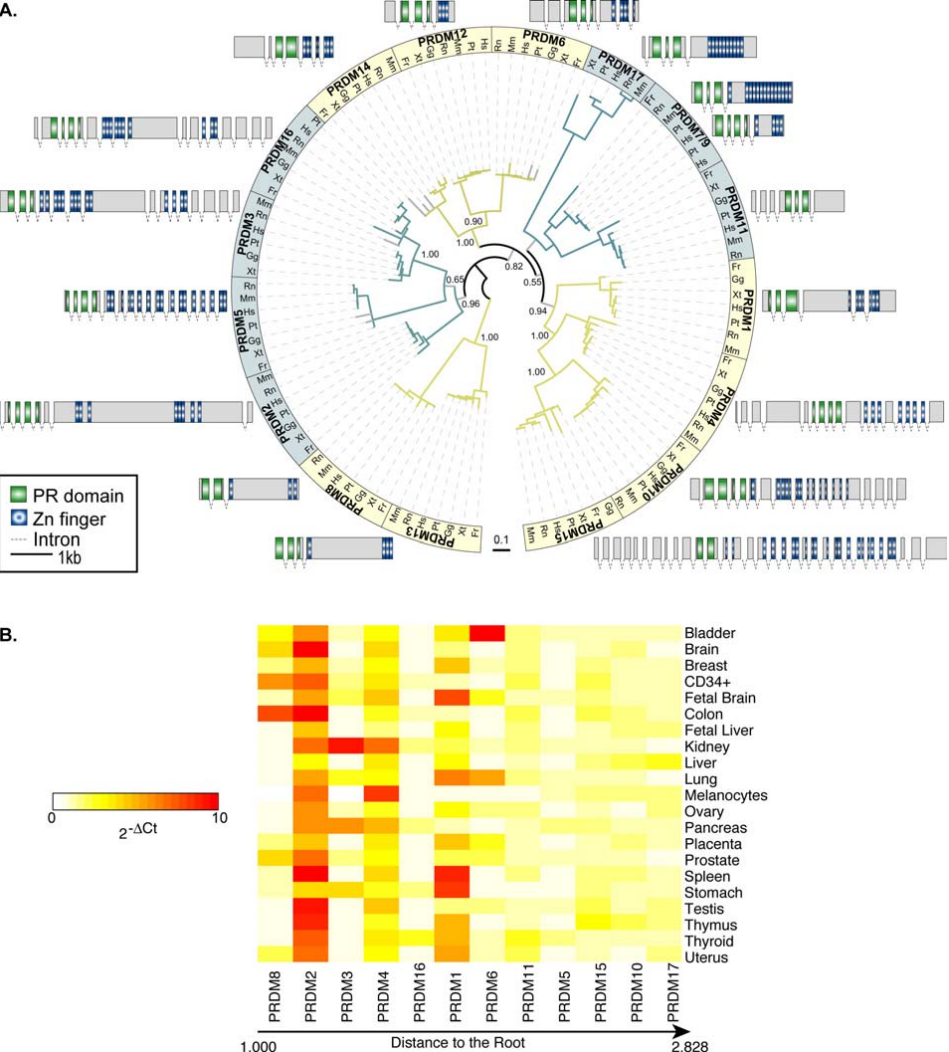
**Phylogenetic tree and expression patterns of the PRDM genes**. **(A) **Phylogenetic tree of the PRDM genes in vertebrates. The reported topology is obtained with Maximum Likelihood. The branches supported with a bootstrap lower than 75 are shown in grey. On main bifurcations, the corresponding posterior probability from Bayesian inference is reported (see Methods). Different colours associated to tree branches correspond to the main subfamilies. For each subfamily, the gene structure of human PRDM ortholog is depicted. The scale refers to exons only. The tree image was produced using iTOL [44]. **(B) **Evolutionary speed and gene expression of the human PRDM paralogs. PRDM genes are ordered by increasing evolutionary divergence, calculated as cumulative branch lengths from the tip to the root of the phylogenetic tree. The expression data were measured as the mean values of different assays for each gene (see Methods). The upper limit of the 2^-ΔCt ^values was set to 10. For original values see Additional file 7.

We rebuilt the phylogenetic relationships between PRDM genes based on the multiple alignment of the PR-domain of all PRDM proteins identified in vertebrates (Figure 2A). The trees obtained by applying both Maximum Likelihood and Bayesian methods are topologically comparable and well supported, the only exception being the internal relationships within the subfamily composed of PRDM7/9, PRDM11 and PRDM17. Overall, five subfamilies are clearly identifiable and the most divergent member is PRDM17. Despite the large variability in terms of gene structure and size among human PRDM paralogs, genes lying in sister branches of the tree maintain similar gene organization (Figure 2A). Such conservation partially reflects similar splicing patterns, as in the case of the subfamily composed of *PRDM2*, *3*, *16*. All these genes can code for both a full-length protein and an isoform without the PR-domain, which is often overexpressed in cancer [22,23].

We observed interesting correlations between gene divergence and expression patterns. In human, all PRDM genes are generally expressed at very low levels, as assessed by qPCR (see Additional file 2). However, substantial differences are detectable among the 17 paralogs in terms of breadth and levels of expression. By ordering the PRDM proteins according to their evolutionary speed, we observed that less divergent paralogs are more expressed than faster-evolving genes (Figure 2B). This result assesses a negative correlation between rate of sequence evolution and gene expression levels (Pearson correlation, r = -0.705, P = 0.01). In addition, some paralogs, such as *PRDM12, 13, 14, 7 *and *9 *acquire tissue specificity, since the corresponding transcripts are detectable only in very few of the analyzed samples (see Additional file 2). One of the youngest members of the family, *PRDM7*, shows an expression pattern mainly restricted to melanocytes, suggesting a progressive specialization and/or tighter regulation of its functions.

### *PRDM7 *arose from *PRDM9 *duplication and acquired a peculiar splicing pattern

**Figure 3:**
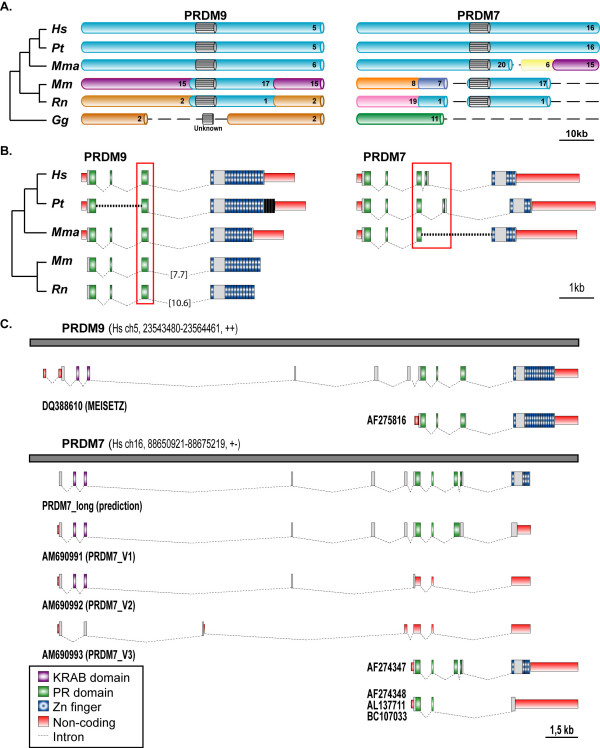
**Syntenic conservation, gene structure, and splicing variants of *PRDM7 *and *PRDM9***. **(A) **Comparison of the syntenic blocks around *PRDM7 *and *PRDM9 *in vertebrates. Each chromosome is depicted in a different colour, except for the genomic regions around the *PRDM7-9 *genes that are all cyan. *PRDM7 *and *9 *are represented as grey blocks. The chromosome number in the corresponding genome is provided. Dashed lines correspond to regions of break of synteny. Abbreviations: *Hs*, Homo sapiens; *Pt*, Pan troglodytes; *Mma*, Macaca mulatta; *Mm*, Mus musculus; *Rn*, Rattus norvegicus; *Gg*, Gallus gallus. **(B) **Gene structure of *PRDM7 *and *PRDM9*. Since for chimp and macaque no mRNA sequences are available, the human *PRDM7 *and *9 *were used as templates for gene predictions. In chimp, the intron putatively gained by *PRDM7 *is composed of eight repeats. In the genomic regions corresponding to chimp *PRDM9*, there are four additional Zn-Fingers, which are reported in black because there is no evidence for their transcription. The dashed lines represent regions of gaps in the genome assembly. In rodents, the last intron is longer and not in scale; the corresponding length is reported in brackets. **(C) **Splicing variants of human *PRDM7 *and *PRDM9*. The grey lines represent the genomic regions of segmental duplication. The corresponding chromosome number, chromosomal coordinates and direction of transcription are given. For *PRDM9*, the splicing variants present in the database are shown. For *PRDM7*, both the database transcripts and the isoforms detected in this study are reported together with an in-silico gene prediction obtained by using the PRDM9 long isoform as template.

To rebuild the possible rearrangements following primate-specific duplication, we compared genetic and genomic features of the primate *PRDM7 *and *PRDM9 *genes with those of their co-ortholog *PRDM7/9 *in non-primates. We observed higher conservation of the genomic block around *PRDM9 *(Figure 3A) and higher similarity of human *PRDM9 *gene structure and protein domain organization with the non-primate co-ortholog (Figure 3B). These results suggest that *PRDM9 *likely retained the features of the ancestral locus, while *PRDM7 *acquired primate-specific features.

**Figure 4:**
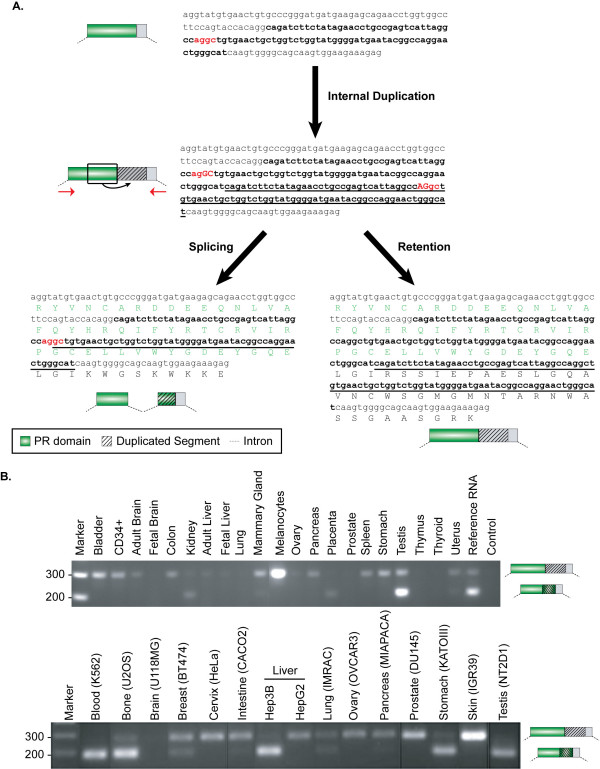
**Gene rearrangements and transcription evidence of *PRDM7***. **(A) **Effects of the internal duplication of ancestral exon 3 on *PRDM7 *splicing. The entire sequence of the ancestral exon 3 is reported; shown are the 89-long segment that undergoes duplication (bold) and the putative cryptic splice sites (red). The duplicon is represented as underlined text. After duplication, the non-canonical splice site (GC-AG) can be activated leading to intron splicing. The entire region can also be retained into the transcript resulting in a protein with no Zn-Fingers due to the introduction of a frameshift. The region in between the two red arrows was amplified in a variety of normal and tumoral samples, as reported in the panels (B). **(B) **RT-PCR analysis of exon 3 in normal tissues and cancer cell lines. The upper panel reports amplifications in normal samples, while the lower in cancer cell lines. We verified by sequence analysis that the upper band corresponds to *PRDM7 *exon 3 retaining the duplicated segment. The lower band can be either exon 3 of *PRDM9 *or exons 3-4 of *PRDM7*, since the two genes are indistinguishable in this region.

After segmental duplication, the new locus experienced massive rearrangements that significantly modified the structure of the resulting *PRDM7 *gene (Figure 3B). In human, the last exon coding for the Zn-Fingers underwent partial deletion and the resulting protein bears only 4 repeats instead of 14 (Figures 1A and 3B). In addition, an 89-nucleotide long segment within ancestral exon 3, which codes for the PR-domain C-terminal part, tandemly duplicated (Figure 4A). This led to the acquisition of a complex pattern of splicing variants (Figure 3C, see below). Interestingly, the duplication of the same 89-nucleotides occurred 8 times in the chimp genome (Figure 3B). However, the possible effect of this repetition on gene splicing is unknown since no chimp mRNA is available.

For human *PRDM9*, both a shorter (AF275816) and a longer (DQ388610) isoform have been reported. In the latter, the corresponding protein add a N-terminal KRAB domain to the usual PRDM architecture (Figure 3C). Meisetz, the mouse ortholog of the long PRDM9 isoform, is able to activate the progression into meiosis through the trimethylation of the lysine 4 on histone H3 [16,24]. Recently, the KRAB domain of Meisetz has been proposed to be the ancestor of all modern KRAB domains, being traceable in the last common ancestor of vertebrates [25]. In principle the genomic locus of *PRDM7 *could also code for a long isoform with the KRAB domain, but all mRNA sequences so far available corresponded to the short variant. One of them (AF274347) resembles the sequence of the short form of *PRDM9 *implying that the duplication inside exon 3 is coupled with an event of intron gain (Figures 3C and 4A). By using specific primers, we cloned the long isoform of *PRDM7 *and found that it can retain the 89-nucleotide long duplicon within the sequence of the mature mRNA (Figures 3C and 4A). This retention introduces a frameshift, and the resulting protein replaces the Zn-Fingers with an alternative C-terminal region (AM690991, Figure 3C). To assess the frequency of the two PRDM7 isoforms in human tissues, we amplified the corresponding region (Figure 4A) in a variety of RNA samples from normal tissues and cancer cell lines. In most cases, two bands of 289 bp and 200 bp are detectable (Figure 4B). Sequence analysis of the RT-PCR products confirmed that the higher band corresponds to the PRDM7-specific amplicon with retention of the internal duplication. The lower band could correspond to either *PRDM7 *or *PRDM9 *since the two genes have identical sequences in this region. The widespread detection of the isoform with the retained duplicon suggests that this might be a general mechanism to produce an alternative PRDM7 protein without Zn-Fingers. Interestingly, in mouse two isoforms of the *Meisetz *gene generated by alternative splicing also lack the Zn-Finger repeats and code for a protein with only the KRAB and the PR domains [16]. By using several combinations of primers, we identified other long isoforms of *PRDM7 *with alternative patterns of exon retention (AM690992 and AM690993, Figure 3C), but we did not retrieve the long isoform coding for the Zn-Fingers. One explanation could be that the long isoform of *PRDM7 *with the Zn-Fingers is not produced. Another possibility is that it is extremely rare, being detectable only in specific tissues and/or cellular phases. This is the case of *Meisetz*, which is only expressed in fetal germ lines entering meiotic prophase [16].

## Conclusion

The PRDM gene family of putative tumor suppressors constitutes an interesting example of vertebrate-specific expansion of transcriptional regulators. Except for *PRDM11*, all PRDM genes code for a variable number of C2H2 Zn-Finger repeats and, although the precise molecular function is mostly unknown, the ability to bind DNA seems to be a common feature. In addition, some of the PRDM genes control transcription through either direct or indirect histone methylation [16,18,19,26]. Thus, rebuilding the progressive specialization of the PRDM ancestral function in different cell types and/or in diverse stages of development may yield general conclusions concerning paralog differentiation of transcriptional regulators in vertebrates.

PRDM genes show an inverse relationship between sequence divergence and expression level. Paralogs that evolve slowly, such as *PRDM8, 2*, and *3 *tend to be more expressed than faster-evolving genes like *PRDM15, 10 *and *17 *(Figure 2B). Although there is not a straightforward biological explanation for it [27,28], this inverse correlation reflects an asymmetrical evolution of PRDM paralogs and is compatible with their progressive sub- and neo-functionalization [7,8]. Both slowly (e.g. PRDM2) and fast-evolving (e.g. PRDM6) paralogs show methyltransferase activity, which implies the repartition of the putative ancestral function among several duplicates. Signs of neo-functionalization are also detectable, such as the different roles of PR domain in histone methylation and the specific involvement of *PRDM9 *in meiotic progression [16,24]. The acquisition of novel functions is particularly evident in the primate-specific gene. As most newborn genes [29,30], *PRDM7 *underwent remarkable structural rearrangements. Some modifications, such as the reduction of encoded Zn-Fingers, are recurrent within the PRDM family (Figure 1A), while the internal duplication of ancestral exon 3 is unusual for at least two reasons. First, the region undergoing duplication codes for the C-terminal part of the PR-domain, which is the only module always present in PRDM proteins. Usually, such well-conserved regions are under strong selective constraints that prevent harmful modifications. Second, the ancestral 89-nucleotide long segment does not contain any repetitive element to justify duplication through replication slippage and/or non-homologous recombination. Thus, the internal duplication seems to reflect adaptation of *PRDM7 *to novel functions. The effect of the duplication on *PRDM7 *splicing may constitute the first evidence for such adaptation. The duplication of a cryptic splice site (AG-GC) in the ancestral sequence results in the acquisition of a non-canonical splice site (GC-AG). This site can activate the splicing of an 89-nucleotide long intron partly deriving from the ancestral sequence and partly from the duplicon (Figure 4A). Such mechanism for intron gain was theoretically described by Rogers in 1989 [31], but has so far been observed very rarely [32,33] and never in human genes. The resulting mature mRNA encodes for a protein with the usual domain architecture of PRDM genes (PR domain and Zn-Fingers). We found that the duplicated segment can be alternatively retained into the mature mRNA and eventually encodes for a frameshifted protein without Zn-Fingers (Figure 3C). Usually, the introduction of a frameshift is a dangerous event for a gene, and indeed mechanisms such as nonsense-mediated decay have evolved to prevent the generation of harmful proteins [34]. However, a few cases of genes duplicated in vertebrates that use frameshift as a mechanism to diversify their function have been previously reported [35]. The majority of these frameshifts originated through alternative splicing able to activate a different reading frame [35]. In the case of *PRDM7*, instead, the frameshift arose through the duplication of an internal sequence. As for the other reported cases [35], also the mRNA transcribed by the *PRDM7 *gene is not a typical target for mRNA decay. It is around 2000 nucleotide long and can code for a protein with KRAB and PR domains and a C-terminal region of around 100 residues before encountering a stop codon (Figure 3C). The isoform with the retention is detectable in most of the tested samples, and it is particularly abundant in melanocytes and melanoma cell lines (Figure 4B). Although more data are needed to confirm actual functional differences, one possible consequence of the lack of Zn-Fingers could be a modified cellular localization. The PRDM9 isoforms without Zn-Fingers are indeed not able to reach cell nucleus [16]. In addition, the alternative C-terminal region of PRDM7 shows no similarity to any sequence so far reported in sequence databases. It would be interesting to check whether this region is associated to the acquisition of any primate-specific function of the corresponding protein.

## Methods

### Detection of PRDM orthologs

To avoid the occurrence of aspecific hits due to the large number of Zn-Fingers, we only used the N-terminal portion of the PRDM proteins for our analysis. As a first scan of the presence of the PRDM genes in the 3 domains of life, we used each human PRDM protein as query for BLASTp [36] on the non-redundant database. To further confirm the presence of PRDMs only in metazoans, we searched the SMART database [37] for the entire repertoire of proteins with the same domain architecture. Finally, we searched the yeast genomes (sacSer1) using the 17 human PR domains as queries for tBLASTn [36]. None of these analyses returned a significant hit for the PRDM genes in non-metazoan genomes.

We used different strategies to derive the orthologs of the human PRDM genes in other 13 metazoans (*Pan troglodytes*, *Macaca mulatta*, *Mus musculus*, *Rattus norvegicus*, *Gallus gallus*, *Xenopus tropicalis*, *Takifugu rubripes*, *Ciona intestinalis*, *Strongylocentrotus purpuratus, Drosophila melanogaster*, *Drosophila pseudoscura*, *Caenorhabditis elegans*, and *Caenorhabditis briggsae*), in order to conduct an orthology assignment as complete as possible. We started by extracting the reciprocal best hits for each PRDM proteins from the output of all all-against-all pairwise BLASTp [36] between human proteins and the protein set of each other species (RefSeq Release 17, April 2006; WormPep v.150; FlyBase 4.3). From the obtained results, we excluded the hits matching on the same chromosomal locus representing likely splice variants or mispredictions. To further enlarge the set of orthologs, we used the sequences of each human PR domain as queries for tBLASTn [36] against the different genome assemblies (human: hg18; chimp: panTro2; macaque: rheMac2; mouse: mm8; rat: rn4; chicken: galGal3; frog: xenTro2; fugu: fr1; ciona: ci2; sea urchin: Spur_0.5; fly: dm2; *D. pseudoscura*: dp4; worm: ce2; *C. briggsae*: cb1). The genomic region corresponding to each best hit was extracted and used for the complete gene prediction with GeneWise [38]. Each predicted sequence was finally blasted back on the human protein set to confirm the orthology relationship through the best reciprocal method. In the cases where the ortholog was still missing, we defined the syntenic blocks around the PRDM genes in the UCSC human genome browser by identifying the genes at their 5' and 3'. We then extracted the corresponding regions in the target genome, and predicted the putative ortholog using GeneWise [38] and Blast2Gene [39]. The complete set of the putative orthologs is reported in the Additional file 1.

### Concatenated alignment and tree reconstruction

We based the evolutionary reconstruction of the PRDM family on the multiple alignment of the amino acidic sequence of the PR-domains. These are, together with the Zn-fingers, the only regions common to all members. Zn-fingers are however not suitable for the tree reconstruction given their repetitive nature. From the entire set of orthologs we only considered the sequences from seven vertebrates (Human, Chimp, Mouse, Rat, Chicken, Fugu, and Frog) and, among those, only the sequences for which a complete PR domain was retrievable. The final set of proteins used for the analysis was composed of 109 sequences, with representatives of each PRDM member in all 7 species except for PRDM3 (missing in fugu), PRDM17 (missing in fugu and chicken), and the non-primate PRDM7/9 (missing in chicken and frog).

For each PR domain of the different PRDM orthologous groups, we built a multiple sequence alignment (MSA) using MUSCLE [40], with the maximum number of iterations set to 100. In the resulting seventeen MSAs, the poorly aligned and divergent regions were eliminated with Gblocks [41]. The reduced alignments were then treated as separate profiles and re-aligned using MUSCLE [40]. The resulting global supermatrix is composed of 181 positions (see Additional file 3).

We derived Maximum Likelihood (ML) phylogenetic inferences using PHYML [42], and applying the JTT matrix. Our model of sequence evolution assumed that there were two classes of sites, one class being invariable and the other class being free to change. The rate variation across these sites was assumed to follow a gamma shape distribution calculated using a discrete approximation with eight categories of sites. Support for the hypotheses of relationships was assessed using 100 bootstrap replicates (see Additional file 4).

For the Bayesian inference, we used MrBayes [43] choosing the Jones substitution model and a gamma-shaped variation across the sites with four categories. The analysis was run for 500000 generations sampling the chain every 100 generations. To derive the final tree, we discarded the first 30% of the samples after checking for the convergence of the analysis after 150000 generations (see Additional file 5).

### Screening of PRDM gene expression in human tissues

Total RNAs derived from normal human tissues (bladder, brain, breast, colon, fetal brain, fetal liver, kidney, liver, lung, ovary, pancreas, placenta, prostate, spleen, stomach, testis, thymus, thyroid and uterus) were obtained from Clontech (Mountain View, Cal., USA). In the case of hemopoietic precursor cells and human melanocytes, the RNA was extracted from CD34+ (from healthy donors) purified using standard procedures and from primary melanocyte cultures, respectively.

The expression levels of the PRDM genes were assessed using a custom TaqMan^® ^Low Density Array (Applied Biosystems, Foster City, Cal., USA). For each PRDM gene a number of assays covering different regions of the transcript were used with 18S and GAPDH as reference genes (see Additional file 2). *PRDM12, 13, 14, 7 *and *9 *are expressed only in very few tissues and, therefore, were not considered for further analysis. In order to reduce the technical variability due to the different efficiency of the assays, for all other genes the Ct value of each assay was plotted, and the corresponding trend in the different tissues was assessed (see Additional file 6). For each gene, the mean value only of the assays with a comparable trend was calculated and normalized using the reference genes. Finally the 2^-ΔCt ^was calculated and normalized to the median of expression across each tissue (see Additional file 7). The resulting values were ordered according to the evolutionary divergence of the PRDM genes. We assessed the statistical significance of the negative association between gene expression level and speed of molecular evolution by plotting the logarithm of the maximum expression value for each PRDM gene against the corresponding distance to the root (Pearson correlation, r = -0.705, P = 0.01). To verify that the inverse relationship between expression data and evolutionary divergence was not due to an artifact, we treated the data from each single array in a comparable manner. In all cases comparable trends were obtained (data not shown).

### RT-PCR and cloning of PRDM7-specific transcripts

Reverse transcription for subsequent PCR reactions was performed with Superscript III Reverse Transcriptase (Invitrogen) according to manufacturer's instructions. Amplification of PRDM7 long isoform was done using the high fidelity Phusion DNA polymerase (Fynnzyme) and different combinations of primers located in the 5'UTR and 3' UTR of the gene. Forward primers: PR7_9L-F1 (5'-TTCTAGACAGTCCCAGCACCATGA-3'), PR7L-F1 (5'-ACTCAGGGGCCCTTCCCACA-3'). Reverse primers: PR7L-R1 (5'-TGCAAGTGTGTGGTGATCACGT-3'), PR7L-R2 (5'-GTGTGTGGTGATCACGTTTGTCTTCT-3'), PR7L-R3 (5'-TTACTCATCCTTCCTGCAGAC-3'). A collection of normal tissues and commercial cancer cell lines was screened together with Reference RNA (Clontech). This is a mixture of total RNAs from a collection of adult human tissues, chosen to represent a broad range of expressed genes.

The PCR products were purified on Wizard SV GEL and PCR clean-up system (Promega) and, after overhangs addition with DyNAzyme II DNA polymerase (Fynnzyme), they were cloned into the pCR2.1-TOPO vector and transformed into TOP10 cells (TOPO TA cloning Kit, Invitrogen). Positive clones were screened by PCR using universal primers and sequenced. The region encompassing the duplicated sequence of PRDM7 was amplified using the following set of primers: forward 5'-TCGGCCAACTGGATGAGGTATGT-3' and reverse 5'-TCCCTGCCATGAGCTCTTTCTT-3'.

## Competing interests

The author(s) declares that there are no competing interests.

## Authors' contributions

IF, NM, MA, FDC conceived and designed the experiments. IF and FDC performed sequence and the evolutionary analyses. NM and GS did the experimental work. MA, FDC, DR analyzed the data. FDC wrote the manuscript. All authors read and approved the final form of the manuscript.

## Supplementary Material

Additional file 1 PRDM orthologs in representatives of metazoans. Detailed description of the PRDM orthologs in 13 metazoans (*Homo sapiens*, *Pan troglodytes*, *Macaca mulatta*, *Mus musculus*, *Rattus norvegicus*, *Gallus gallus*, *Xenopus tropicalis*, *Takifugu rubripes*, *Ciona intestinalis*, *Drosophila melanogaster*, *Drosophila pseudoscura*, *Caenorhabditis elegans*, and *Caenorhabditis briggsae*). Click here for file

Additional file 2 Expression data used for the analysis. The reported data represent the Ct values for each PRDM gene in 21 tissues obtained by qPCR. Click here for file

Additional file 3 Multiple alignment used for the phylogenetic reconstruction. Multiple alignment of the vertebrate PRDM orthologs in phylip format. Click here for file

Additional file 4 Tree data obtained with maximum likelihood. Phylogenetic tree of the PRDM genes in vertebrates using maximum likelihood in newick format. Click here for file

Additional file 5 Tree data obtained with Bayesian inference. Phylogenetic tree of the PRDM genes in vertebrates using Bayesian inference in newick format. Click here for file

Additional file 6 Trends of expression with different assays. The plots report the trend of expression of each human PRDM gene in 21 different tissues. Click here for file

Additional file 7 Expression data from qPCR. Original data used for building Figure 2B. Click here for file
