# Supplementary material for: Family expansion and gene rearrangements contributed to the functional specialization of PRDM genes in vertebrates
Source: BMC Evol Biol. 2007 Oct 4;7:187. doi: 10.1186/1471-2148-7-187 (PMC2082429; doi:10.1186/1471-2148-7-187)
Supplement: Additional file 1 — PRDM orthologs in representatives of metazoans. Detailed description of the PRDM orthologs in 13 metazoans (Homo sapiens, Pan troglodytes, Macaca mulatta, Mus musculus, Rattus norvegicus, Gallus gallus, Xenopus tropicalis, Takifugu rubripes, Ciona intestinalis, Drosophila melanogaster, Drosophila pseudoscura, Caenorhabditis elegans, and Caenorhabditis briggsae). [file 1471-2148-7-187-S1.pdf]

Additional file1: PRDM orthologs in representatives of metazoans.

Gene	Hs (17)		Pt (17)		Mmu (17)		Mm (16)		Rn (16)		Gg (16)		Xt (16)		Fr (15)	
	Identifier	ZnF	Identifier	ZnF	Identifier	ZnF	Identifier	ZnF	Identifier	ZnF	Identifier	ZnF	Identifier	ZnF	Identifier	ZnF
**PRDM1**	NP_001189	5	PRDM1_pt	5	PRDM1_mmu	5	NP_031574	5	XP_228320	5	PRDM1_gg	5	BC080145	5	Q75UA3	5
**PRDM2**	NP_056950	8	PRDM2_pt	8	PRDM2_mmu	8	XP_204027	8	Q63755	8	PRDM2_gg	8	PRDM2_xt	8	PRDM2_fr	8
**PRDM3**	NP_005232	10	PRDM3_pt	10	PRDM3_mmu	10	PRDM3_mm	10	PRDM3_rn	10	PRDM3_gg	10	PRDM3_xt	8	PRDM3_fr	10
**PRDM4**	NP_036538	7	PRDM4_pt	7	PRDM4_mmu	7	NP_857633	7	NP_579846	7	PRDM4_gg	7	NP_001072257	7	PRDM4_fr	7
**PRDM5**	NP_061169	16	PRDM5_pt	16	PRDM5_mmu	14	NP_081823	15	PRDM5_rn	13	XP_420628	16	PRDM5_xt	15	PRDM5_fr	16
**PRDM6**	XP_932740	4	PRDM6_pt	4	PRDM6_mmu	4	NP_001028453	4	PRDM6_rn	4	PRDM6_gg	4	PRDM6_xt	4	PRDM6_fr	*
**PRDM7**	NP_443722	4	PRDM7_pt	3	PRDM7_mmu	3	Meisetz	13	XP_344848	11	PRDM7/9_gg	13	PRDM7/9_xt	*	PRDM7/9_fr	11
**PRDM8**	NP_064611	3	PRDM8_pt	3	PRDM8_mmu	3	NP_084223	2	XP_223205	1	PRDM8_gg	*	NP_001039151	2	PRDM8_fr	2
**PRDM9**	NP_064612	14	PRDM9_pt	14	PRDM9_mmu	10	Meisetz	13	XP_344848	11	PRDM7/9_gg	13	PRDM7/9_xt	*	PRDM7/9_fr	11
**PRDM10**	NP_955470	10	PRDM10_pt	9	PRDM10_mmu	10	XP_356146	10	XP_576373	9	PRDM10_gg	10	PRDM10_xt	10	PRDM10_fr	10
**PRDM11**	NP_064614	---	PRDM11_pt	---	PRDM11_mmu	---	XP_921193	---	XP_215777	---	PRDM11_gg	---	PRDM11_xt	---	PRDM11_fr	---
**PRDM12**	NP_067632	3	PRDM12_pt	3	PRDM12_mmu	3	XP_355325	3	PRDM12_rn	3	XP_415465	3	PRDM12_xt	*	PRDM12_fr	3
**PRDM13**	NP_067633	4	PRDM13_pt	4	PRDM13_mmu	4	XP_889460	4	XP_345510	3	PRDM13_gg	*	PRDM13_xt	4	PRDM13_fr	4
**PRDM14**	NP_078780	6	PRDM14_pt	6	PRDM14_mmu	6	XP_918536	6	PRDM14_rn	3*	PRDM14_gg	6	PRDM14_xt	6	PRDM14_fr	3*
**PRDM15**	NP_071398	17	PRDM15_pt	2*	PRDM15_mmu	17	XP_912670	17	XP_239504	16	XP_416740	17	PRDM15_xt	17	PRDM15_fr	15
**PRDM16**	NP_071397	10	PRDM16_pt	10	PRDM16_mmu	10	NP_081780	10	PRDM16_rn	*	XP_417551	5	PRDM16_xt	10	PRDM16_fr	10
**PRDM17**	NP_079017	10	PRDM17_pt	10	PRDM17_mmu	10	XP_929048	10	XP_230283	10	PRDM17_gg	8	PRDM17_xt	10	---	---

Ci (2)		Dm (3)		Dp (3)		Ce (2)		Cb (2)	
Identifier	ZnF	Identifier	ZnF	Identifier	ZnF	Identifier	ZnF	Identifier	ZnF
AK117075	5	CG5249 (PRDM1; chr. 3L)	5	PRDM_dp_chrXR (PRDM1)	5	F25D7.3 (PRDM1, chr. I)	5	CBG02201 (PRDM1)	5
AK114889	7	CG9817 (PRDM5; chr. X)	6	PRDM_dp_chrXL (PRDM5)	6	T21B10.5 (chr. II)	--	Cb25.fpc0058	--
---		CG31753 (Hamlet, chr. 2L)	9	Hamlet_dp_chr4	9	---	--	---	--

Legend: Where present, the accession numbers of the corresponding proteins in the database are reported. The sequences assembled directly from our predictions, have been named using the PRDM protein number followed by the abbreviation of the species. Human PRDM7 and PRDM9 are co-orthologs of the same protein in non-primate species. In the case of PRDM17 in fugu only the Zn-Finger region could be predicted. Abbreviations: Hs=*Homo sapiens*; Pt=*Pan troglodytes*; Mmu=*Macaca mulatta*; Mm=*Mus musculus*; Rn=*Rattus norvegicus*; Gg=*Gallus gallus*; Xt=*Xenopus tropicalis*; Fr=*Fugu rubripes*. *: The correct number of Zinc-Fingers could not be predicted due to the presence of gaps in the corresponding genome assembly.
