# Supplementary material for: Family expansion and gene rearrangements contributed to the functional specialization of PRDM genes in vertebrates
Source: BMC Evol Biol. 2007 Oct 4;7:187. doi: 10.1186/1471-2148-7-187 (PMC2082429; doi:10.1186/1471-2148-7-187)
Supplement: Additional file 6 — Trends of expression with different assays. The plots report the trend of expression of each human PRDM gene in 21 different tissues. [file 1471-2148-7-187-S6.pdf]

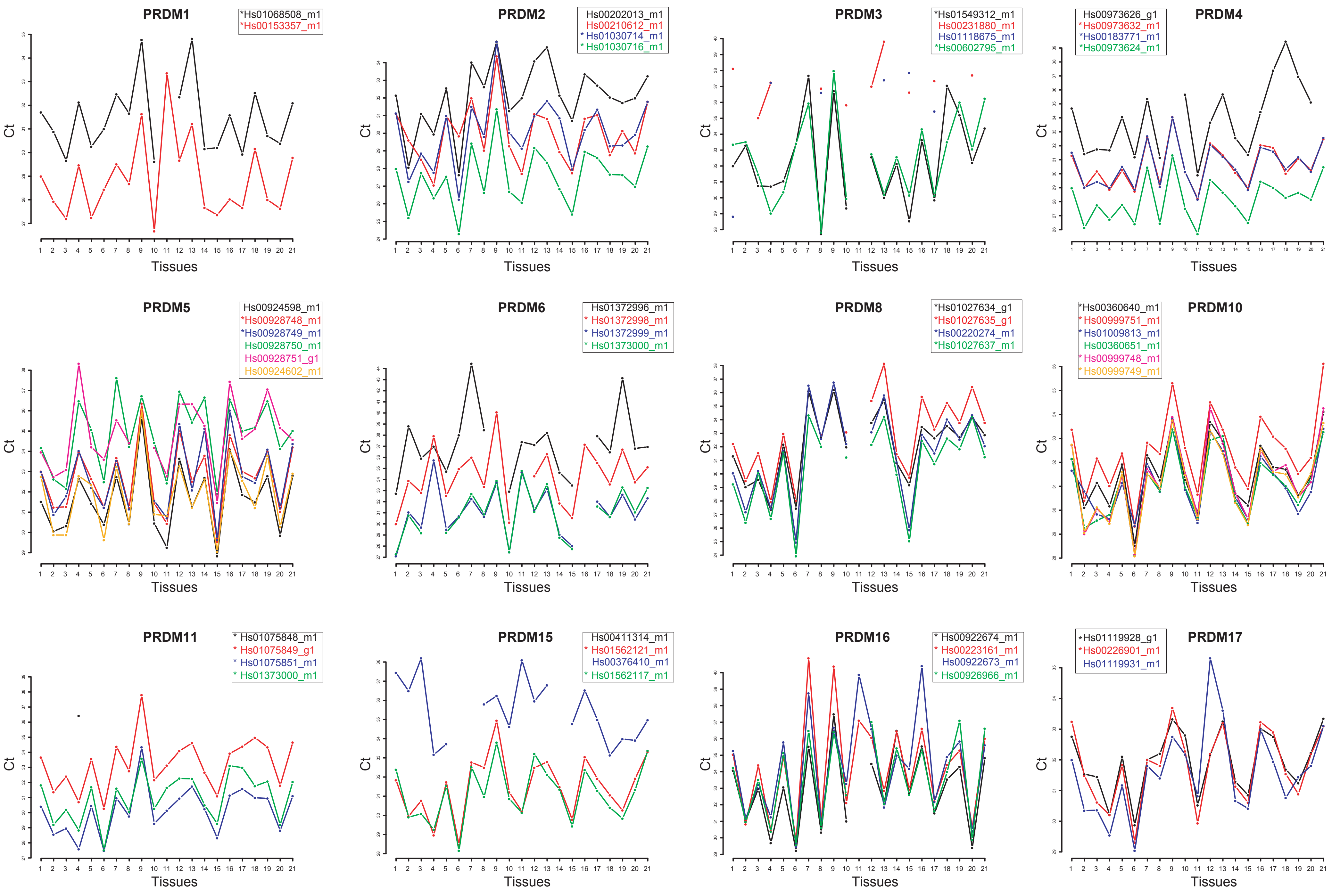
PRDM1
*Hs01068508_m1
*Hs00153357_m1
PRDM2
Hs00202013_m1
Hs00210612_m1
* Hs01030714_m1
* Hs01030716_m1
PRDM3
*Hs01549312_m1
Hs00231880_m1
Hs01118675_m1
*Hs00602795_m1
PRDM4
Hs00973626_g1
*Hs00973632_m1
*Hs00183771_m1
*Hs00973624_m1
PRDM5
Hs00924598_m1
*Hs00928748_m1
*Hs00928749_m1
Hs00928750_m1
Hs00928751_g1
Hs00924602_m1
PRDM6
Hs01372996_m1
* Hs01372998_m1
* Hs01372999_m1
* Hs01373000_m1
PRDM8
*Hs01027634_g1
*Hs01027635_g1
*Hs00220274_m1
*Hs01027637_m1
PRDM10
*Hs00360640_m1
*Hs00999751_m1
*Hs01009813_m1
Hs00360651_m1
*Hs00999748_m1
*Hs00999749_m1
PRDM11
* Hs01075848_m1
* Hs01075849_g1
* Hs01075851_m1
* Hs01373000_m1
PRDM15
Hs00411314_m1
* Hs01562121_m1
Hs00376410_m1
* Hs01562117_m1
PRDM16
* Hs00922674_m1
* Hs00223161_m1
Hs00922673_m1
* Hs00926966_m1
PRDM17
*Hs01119928_g1
*Hs00226901_m1
Hs01119931_m1
Ct
Tissues

Legend to additional file 6:

For each gene, the Ct values deriving from each qPCR experiment were plotted to assess the corresponding trend in all examined tissues. Every assay is depicted with a different colour. The starred assays are the one taken for the analysis. Tissue legend: 1: Bladder; 2: Brain; 3: Breast; 4: CD34+; 5: Colon; 6: Fetal Brain; 7: Fetal Liver; 8: Kidney; 9: Liver; 10: Lung; 11: Melanocytes; 12: Ovary; 13: Pancreas; 14: Placenta; 15: Prostate; 16: Spleen; 17: Stomach; 18: Testis; 19: Thymus; 20: Thyroid; 21: Uterus.
